# Implementing a digital intervention for managing uncontrolled hypertension in Primary Care: a mixed methods process evaluation

**DOI:** 10.1186/s13012-021-01123-1

**Published:** 2021-05-26

**Authors:** Kate Morton, Laura Dennison, Rebecca Band, Beth Stuart, Laura Wilde, Tara Cheetham-Blake, Elena Heber, Joanna Slodkowska-Barabasz, Paul Little, Richard J. McManus, Carl R. May, Lucy Yardley, Katherine Bradbury

**Affiliations:** 1 Academic Unit of Psychology, University of Southampton, Southampton, UK; 2 Health Sciences, University of Southampton, Southampton, UK; 3 Primary Care Research, University of Southampton, Southampton, UK; 4 Centre for Intelligent Healthcare, Faculty of Health and Life Sciences, Coventry University, Coventry, UK; 5 NIHR Evaluation, Trials and Studies Coordinating Centre, University of Southampton, Southampton, UK; 6 GET.ON Institut, Hamburg, Germany, & University of Southampton, Southampton, UK; 7 Nuffield Department of Primary Care Health Sciences, University of Oxford, Oxford, UK; 8 Faculty of Public Health and Policy, London School of Hygiene and Tropical Medicine, London, UK; 9 School of Psychological Science, University of Bristol, Bristol, UK

**Keywords:** Mixed methods, Process evaluation, Hypertension, Blood pressure, Normalisation Process Theory, Digital intervention

## Abstract

**Background:**

A high proportion of hypertensive patients remain above the target threshold for blood pressure, increasing the risk of adverse health outcomes. A digital intervention to facilitate healthcare practitioners (hereafter practitioners) to initiate planned medication escalations when patients’ home readings were raised was found to be effective in lowering blood pressure over 12 months. This mixed-methods process evaluation aimed to develop a detailed understanding of how the intervention was implemented in Primary Care, possible mechanisms of action and contextual factors influencing implementation.

**Methods:**

One hundred twenty-five practitioners took part in a randomised controlled trial, including GPs, practice nurses, nurse-prescribers, and healthcare assistants. Usage data were collected automatically by the digital intervention and antihypertensive medication changes were recorded from the patients’ medical notes. A sub-sample of 27 practitioners took part in semi-structured qualitative process interviews. The qualitative data were analysed using thematic analysis and the quantitative data using descriptive statistics and correlations to explore factors related to adherence. The two sets of findings were integrated using a triangulation protocol.

**Results:**

Mean practitioner adherence to escalating medication was moderate (53%), and the qualitative analysis suggested that low trust in home readings and the decision to wait for more evidence influenced implementation for some practitioners. The logic model was partially supported in that self-efficacy was related to adherence to medication escalation, but qualitative findings provided further insight into additional potential mechanisms, including perceived necessity and concerns. Contextual factors influencing implementation included proximity of average readings to the target threshold. Meanwhile, adherence to delivering remote support was mixed, and practitioners described some uncertainty when they received no response from patients.

**Conclusions:**

This mixed-methods process evaluation provided novel insights into practitioners’ decision-making around escalating medication using a digital algorithm. Implementation strategies were proposed which could benefit digital interventions in addressing clinical inertia, including facilitating tracking of patients’ readings over time to provide stronger evidence for medication escalation, and allowing more flexibility in decision-making whilst discouraging clinical inertia due to borderline readings. Implementation of one-way notification systems could be facilitated by enabling patients to send a brief acknowledgement response.

**Trial registration:**

(ISRCTN13790648). Registered 14 May 2015.

**Supplementary Information:**

The online version contains supplementary material available at 10.1186/s13012-021-01123-1.

Contributions to the literature

- This mixed-methods study explored the implementation process for practitioners using a digital intervention shown to be effective for lowering blood pressure.
- Practitioners showed moderate adherence to escalating medication based on home readings.
- Diverse perceptions of implementing medication escalations when prompted were revealed, with some practitioners perceiving that the intervention facilitated appropriate medication escalation whilst a few described low perceived necessity and/or concerns about patient risk.
- Adherence to remotely notifying patients of medication escalation was low.
- Definitions of appropriate inaction could facilitate future implementation of interventions addressing clinical inertia.

## Background

Clinical inertia occurs when healthcare practitioners (hereafter ‘practitioners’) do not intensify patients’ medication despite raised readings during a consultation [1] and contributes to sub-optimal hypertension control [2]. Clinical inertia can be attributed to reluctance to base decisions on one-off clinic readings, low confidence in medication effectiveness, concerns about side effects or patient reluctance to escalate medication, and lack of time during appointments [3].

A digital intervention (called HOME BP) was developed using Social Cognitive Theory (SCT) [4] to target clinical inertia and optimised using the person-based approach [5–7]. A patient component sought to increase self-efficacy to self-monitor blood pressure and positive outcome expectancies about receiving medication increases when needed, and a practitioner component targeted self-efficacy to escalate medication based on patients’ home readings, in line with a plan created in advance with each patient [6–9]. This personalised three-step medication plan was theorised to reduce the risk of clinical inertia arising at the time of medication escalation, based on procedures from non-digital interventions which successfully reduced blood pressure without adverse outcomes such as increased side-effects or patient anxiety or dissatisfaction [10, 11]. The HOME BP digital intervention provided an open-text box each time a medication escalation was recommended, to encourage patients to send their practitioner a message if they wanted to share any concerns or additional information. Practitioners could also email their patient through the intervention and received feedback on whether or not the patient reported implementing a medication escalation. This ensured both practitioners and patients remained in close contact and if either had any concerns about the medication escalation, the recommendation could be overridden. HOME BP was found to successfully increase antihypertensive medication escalations in Primary Care, and led to significant reductions in systolic blood pressure [12]. A qualitative process evaluation of patients’ experiences of using HOME BP showed that perceived benefits included reassurance that uncontrolled hypertension was being addressed, whilst worry about health and fitting self-monitoring into the day could be burdens for patients [13].

To date, no theory-informed mixed-methods process evaluations have been conducted of interventions addressing clinical inertia in hypertension, which limits our understanding of how and why such interventions might be effective. Process evaluations enable important insights into the implementation of an intervention, mechanisms of change, and contextual factors [14], which can help inform how best to optimise the intervention for further implementation, and how to adapt the intervention to new contexts This mixed-methods process evaluation aimed to explore practitioners’ adherence and perceptions of implementing the HOME BP intervention in Primary Care, the possible change mechanisms, and any contextual factors that facilitated or hindered implementation. Normalisation Process Theory [15] was used to interpret the implications for normalising the intervention in Primary Care.

## Methods

### Design

**Fig. 1:**
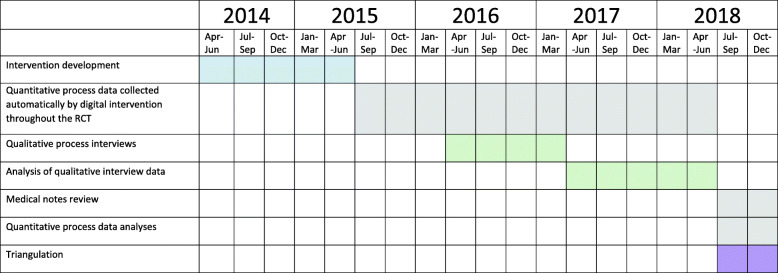
Timeline for the nested process evaluation within the RCT

This was a mixed-methods process study nested within a randomised controlled trial (RCT); see Fig. 1.

Randomisation was stratified by Practice, so each practitioner had experience of delivering usual care and using HOME BP. Quantitative intervention usage data and measures of adherence were collected from all practitioners in the trial (*n* = 125). Qualitative interviews were conducted with a sub-sample of practitioners during the trial (*n* = 27).

The study used a parallel mixed-methods design in that the quantitative and qualitative data were collected concurrently during the RCT, with the exception of quantitative data such as medication escalations which could only be collected after the RCT had finished. The quantitative data and qualitative data were analysed in isolation and then the findings compared to interpret to what extent they converged, diverged, or complemented one another [16]. Both types of data were treated with equal importance, in line with a triangulation design [17].

The study was approved by the University of Southampton and NHS Research Ethics committees (15/SC/0082). The GRAMMS checklist for mixed methods research [18] and StaRI checklist for implementation studies [19] were used to ensure comprehensive reporting (Additional file 1).

### Intervention and proposed mechanisms of action

HOME BP was an online intervention for patients and practitioners which aimed to reduce uncontrolled hypertension in Primary Care [9]. It was trialled at a time when controlling blood pressure to a threshold below 150/90 mmHg was an audit target of the national Quality and Outcomes Framework in UK General Practice [20], and a move towards patient self-management was a priority for chronic conditions [21].

The intervention procedures are described with reference to behaviour change theory. Practitioners completed a mandatory online training session of approximately 20–30 min tailored to their role (prescriber; a GP or nurse prescriber, or supporter; a nurse or healthcare assistant). At some Practices, a prescriber chose to perform both roles, acting as a ‘prescriber-supporter’. The training aimed to increase practitioners’ positive outcome expectancies by showing that intervention procedures were evidence-based and acceptable to patients, particularly how escalating medication in response to average home readings according to a threshold could improve blood pressure control without increasing side effects [10]. Prescribers were then trained to create a three-step plan for medication escalation with the patient. Worked examples were provided to increase self-efficacy. Supporter training explained how to send monthly support emails to patients using pre-written templates (Additional file 2) to promote ongoing engagement in self-monitoring blood pressure and how to use the CARE approach (Congratulate, Ask, Reassure, Encourage) [6] during optional support appointments. The CARE approach was developed to help practitioners provide patient-centred care alongside digital interventions without the need for specialist skills in behaviour change [22, 23]. The training included examples of using CARE during conversations with patients, and evidence to support acceptability of CARE to patients, to increase self-efficacy and outcome expectancies.

Patients independently completed online training at home to raise self-efficacy to self-monitor blood pressure (for more details, see [13]). Emails were then sent to prescribers each month with the patient’s average blood pressure readings over 7 days, and any recommended action according to an algorithm based on the NICE guidelines for home readings (Additional file 3) [9]. Table 1 describes the target behaviours for prescribers and supporters.

**Table 1:** HOME BP intervention procedures for prescribers and supporters

Practitioner	Target behaviour	Description
Prescriber	Planning medication escalations	At a baseline consultation, prescribers planned three potential consecutive medication escalations which they would initiate if the patient’s average blood pressure was raised for two consecutive months during the trial.
	Changing medication in response to recommendations	When patients’ average blood pressure readings were above-target for two consecutive months, prescribers received an automated email recommending they make the next planned medication escalation (Additional file 2). When patients had a one-off very high or very low reading, the automated email recommended a clinical review. The patient could email their prescriber via the intervention in the case of raised blood pressure readings or after a recent medication escalation. Prescribers could reply to patients via email using the HOME BP programme.
	Notifying patient of medication escalation via remote communication	A template letter was provided for practitioners to send patients, asking them to pick up the prescription.
Supporter	Providing remote support	Supporters were prompted by automated email to send monthly support emails to patients using pre-written templates (Additional file 3). These templates were designed to keep patients motivated to continue self-monitoring their blood pressure and engaging in any healthy lifestyle changes (an optional add-on). Supporters could also send ad hoc emails to patients. These could be supporter-initiated (e.g. congratulating them on well-controlled readings or asking about a new medication) or patient-initiated (e.g. to respond to emails sent from patients via HOME BP using the ‘Ask the Nurse’ function).
	Providing in-person support using the CARE approach	In-person support was designed to be minimal, but patients were offered optional appointments to help learn how to use the blood pressure monitor, and to support them in choosing a healthy lifestyle change.

**Fig. 2:**
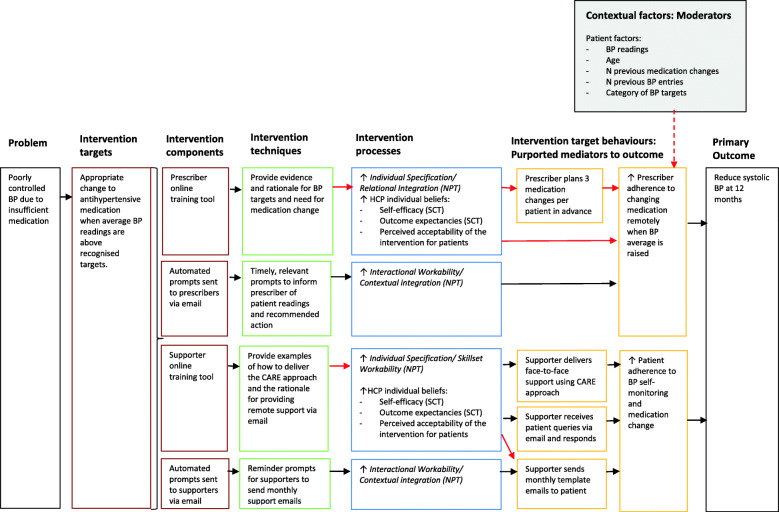
HOME BP logic model showing hypothesised mechanisms of change

Figure 2 shows the logic model representing hypothesised mechanisms of action. This built on the logic model developed during intervention planning [8]. It was hypothesised that the online training would increase practitioners’ self-efficacy and outcome expectancies regarding escalating medication, in line with SCT [4], and promote perceived acceptability of the intervention for patients [24]. In turn, these beliefs were theorised to relate to adherence to the target behaviours. Patient factors (such as blood pressure readings, age, and *n* of previous medication escalations recommended) were theorised to moderate adherence to escalating patients’ medication, based on known reasons for clinical inertia in tele-monitoring interventions [25, 26].

NPT [15] helped elucidate which mechanisms of implementation the intervention techniques were targeting. NPT proposes that four mechanisms influence the incorporation of an intervention into everyday practice: *Coherence* (understanding and making sense of a new practice), *Cognitive Participation* (organisation of roles and engagement in set-up of a practice), *Collective Action* (implementing the workflow of a new practice), and *Reflective Monitoring* (evaluation of the value of a practice and plans for ongoing engagement). The online training for practitioners aimed to increase their *Individual Specification* (a component of *Coherence)* by explaining the rationale and evidence for the intervention processes and to increase *Skillset Workability* (a component of *Collective Action)* by showing how to implement the intervention in practice. The email prompts to escalate medication or send patient support emails were theorised to act on *Interactional Workability* and *Contextual Integration* (both components of *Collective Action*) by facilitating action using a procedure compatible with existing practice.

Relationships could only be tested in this process evaluation if the contextual factors and target behaviour were captured quantitatively, shown in red in Fig. 2. The qualitative interviews explored all aspects of the intervention.

### Data collection and measures

#### Quantitative

SCT [4] and evidence from previous hypertension intervention trials [25, 26] informed the present process evaluation, enabling the selection of measures to capture mechanisms anticipated to lead to change [27], and contextual factors anticipated to influence adherence. Table 2 shows the data sources contributing to each of the three process evaluation themes: implementation, mechanisms and context, as well as the timepoint at which each data source was collected. Self-report questionnaires measuring self-efficacy, outcome expectancies, and perceived acceptability of the intervention to patients were completed before and after the online training to explore mechanisms (Additional file 4). Emails sent to patients via the intervention were collected automatically by the intervention and a review of patients’ medical notes extracted medication changes to explore implementation. Patient age and blood pressure readings were captured by the intervention to explore contextual factors.

**Table 2:** Quantitative data for the process evaluation

Process evaluation theme	Variable	Data source	Timepoint
Implementation	Planned medication escalations	Patient medical notes	Post 12-month follow-up
	*N* of medication escalation recommendations per prescriber	Objective data automatically recorded by intervention software	Throughout study
	*N* and dates of medication escalations initiated	Patient medical notes	Post 12-month follow-up
	Method for contacting patients re medication escalation	Patient medical notes	Post 12-month follow-up
	*N* of support emails sent to patients via HOME BP	Objective data automatically recorded by intervention software	Post 12-month follow-up
Mechanisms	Self-efficacy to implement the intervention procedures	3-item self-report questionnaire (Additional file 4)	Pre and post training module at baseline
	Outcome expectancies about the intervention	6-item self-report questionnaire (Additional file 4)	Pre and post training module at baseline
	Perceived acceptability of the intervention for patients	3-item self-report questionnaire (Additional file 4)	Pre and post training module at baseline
Contextual factors	Systolic and diastolic blood pressure readings entered by patient	Objective data automatically recorded by intervention software	Throughout study
	*N* of blood pressure entries and *n* of medication escalation recommendations per patient	Objective data automatically recorded by intervention software	Throughout study
	Patient age	Objective data automatically recorded by intervention software	Baseline
	Patient blood pressure targets: a) Standard (135/85 mmHg) b) Adjusted due to diabetes (135/75 mmHg) c) Adjusted due to age (145/85 mmHg if aged over 80 years)	Objective data automatically recorded by intervention software	Baseline

#### Qualitative

Potential participants were invited to interview by email and provided informed consent by freepost or online. The semi-structured interview schedule (Additional file 5) used open questions to explore practitioners’ experiences and perceptions of the intervention, rather than deductive questions based on the theories anticipated to influence implementation (SCT and NPT). This was in line with an inductive approach to the qualitative analysis, with subsequent interpretation of the findings using SCT and NPT. The interviews were conducted by telephone between March 2016 and April 2017, and GP Practices were reimbursed for participants’ time.

All interviewers were female researchers in Health Psychology at the University of Southampton with previous experience of interviewing (KM, LW, TCB, EH, and JSB). Interviewers were trained by KM using one-to-one sessions to familiarise each interviewer with the interview schedule and the intervention procedures, followed by a practice interview to promote consistency. KM also provided feedback to each interviewer following transcription of their first interview. Each interview was audio-recorded, except in two cases where the technology failed and detailed notes were used in the analysis instead. Verbatim transcriptions were checked by the interviewer.

### Participants

#### Quantitative

All GP Practices which randomised patients to the intervention group were included in the study (*n* = 70/76). The sample of practitioners was determined by the number of GP Practices required to recruit 610 patients [9].

#### Qualitative

Sampling was initially opportunistic, but subsequently purposive sampling was used to target practices with higher numbers of patients in the study and where one practitioner acted as prescriber *and* supporter, informed by concurrent analysis.

### Analysis

#### Quantitative

Adherence rates to indicate implementation fidelity were calculated as follows:

- Mean adherence to planning medication escalations (100% adherence would be three planned escalations per patient).
- Mean adherence to initiating recommended medication escalations (*n* of recommended medication escalations initiated within 28 days/total medication escalations recommended by the intervention). Twenty-eight days was the threshold agreed by two clinicians, which ensured the escalation was made before the next set of blood pressure readings was submitted by the patient.
- Proportion of medication escalations made remotely (email or letter).
- Mean adherence to sending monthly support emails to patients.

Wilcoxon matched pairs tests were used to compare practitioners’ questionnaire scores before and after training, as the data did not meet assumptions for parametric tests. All questionnaire scales were analysed as mean scores as the Cronbach’s alpha indicated good internal consistency (> 0.8), except for the 3-item prescriber scales assessing self-efficacy and perceived acceptability for patients, which were treated as individual items due to a lower Cronbach’s alpha pre-training (*α* = 0.67).

Spearman’s correlations assessed the relationships between questionnaire scores before and after training and adherence to prescribers’ and supporters’ target behaviours. Contextual factors theorised in the logic model to influence adherence to medication escalation (specifically, patient’s mean systolic and diastolic blood pressure reading, age, number of previous recommended medication escalations, number of previous blood pressure entries, and category of blood pressure targets used for the patient—standard, diabetic, or aged over 80) were compared between recommendations adhered to and those not adhered to using Mann-Whitney *U* tests for continuous data and chi squared-tests for categorical data.

#### Qualitative

Interview data were analysed by KM using reflexive thematic analysis in order to inductively explore practitioners’ experiences and perceptions of implementing the intervention [28, 29]. The interview transcripts were read thoroughly to develop familiarity with the data, and then codes were assigned to begin labelling and collating the data in NVivo 10. Initial themes were developed which helped identify common meaning amongst the codes, and these were subsequently reviewed and refined in order to ensure they represented participants’ experiences. During this phase, KM wrote memos about patterns in the data, using the technique from grounded theory [30], which helped to understand possible meaning in the dataset. KM met with LY and KB frequently to discuss the initial coding, generation of themes, reviewing themes, and describing and naming the themes. KB and LY are both health psychologists who brought qualitative expertise as well as detailed understanding of the intervention and target behaviours.

The themes were defined in a coding manual (Additional file 6) and written up as a narrative. The narrative description of the themes was discussed with RM and PL who offered a clinical perspective on the findings. Each theme was subsequently mapped back to the NPT mechanisms to help understand the implications of the findings for implementation. This process was conducted by KM using standard definitions of the NPT mechanisms, with subsequent discussions with co-authors, especially CM.

### Integration

A triangulation matrix was used to integrate findings from the quantitative and qualitative analyses [31]. Some themes developed in the inductive thematic analysis were too broad to map directly to the quantitative findings; therefore, the triangulation matrix extracted qualitative findings at the level of both themes and sub-themes. Summary statements were written for each key finding [32] and triangulated to establish whether they were in agreement, partial agreement (the two findings complemented one another), dissonant (the findings conflicted), or silent (only one data source contributed) [31, 33].

## Results

Table 3 shows the sociodemographic characteristics of the sample. The quantitative analyses included 125 practitioners, comprised of 62 prescribers, 58 supporters, and 5 prescriber-supporters who performed both roles. Quantitative data were collected from all 125 practitioners in the RCT, except the baseline questionnaires which were completed by 124/125 (99%). A sub-sample of 44 practitioners (35%) were invited to participate in qualitative process interviews, and 27 agreed to take part (61% acceptance rate, 22% of overall sample). The qualitative interview sample was comprised of 13 prescribers (GPs), 11 supporters (7 Practice Nurses, 1 Nurse Prescriber, 2 Healthcare Assistants, and 1 deputy Practice Manager), and 3 prescriber-supporters (Nurse Practitioners). The mean Index of Multiple Deprivation (IMD) of the GP Practices was 7.5 (range 1–10) and 8.0 (range 1–10) for the qualitative and quantitative samples respectively (1 indicates an area lies within the most deprived 10% in the UK, and 10 indicates the least deprived 10%). The mean IMD for GP Practices who were invited to interviews but did not participate was 7.8.

**Table 3:** Sociodemographic and study details of qualitative and quantitative samples

	Participants providing qualitative data (*n* = 27)			Participants providing quantitative data (*n* = 125)		
	Prescribers	Supporters	Prescriber-supporters	Prescribers	Supporters	Prescriber-supporters
*n*	13	11	3	62	58	5
Gender	5 female (38%)	10 female (91%)	3 female (100%)	22 female (35%)	55 female (95%)	3 female (60%)
Mean *n* of patients in intervention group at each Practice (range)	5 (2–10)	5 (2–8)	7 (2–10)	4.3 (− 1–12)	4.4 (1–12)	6.2 (2–10)
Mean *n* of weeks from randomisation of first participant to time of interview (range)	29 weeks (17–54)	27 weeks (20–43)	20 weeks (16–24)	N/A		
Mean duration of interview (range)	26:14 (14–37 min)	29:02 (11–62 min)	43:19 (37–53 min)	N/A		
Mean *n* of recommendations for medication escalation received by prescriber at point of interview (range)	3 (0–7)	N/A	3 (1–4)	N/A		

### Implementation

The themes developed in the qualitative analysis are shown in Table 4, whilst adherence rates to each target behaviour are shown in Table 5.

**Table 4:** Themes developed from the thematic analysis, mapped on to NPT constructs

Theme	Sub-theme	Definitions	NPT construct
Ease or burden of implementing HOME BP		Perceptions about how well the digital intervention fits with current roles	Coherence (Individual Specification)
		How tasks were implemented with colleagues	Collective action (Interactional Workability)
Belief in the concept of HOME BP		Perceptions about how the digital intervention fitted with organisational goals or patient outcomes	Coherence (Internalisation)
Supporting patients to manage their own blood pressure	Planning medication escalations	How prescribers adapted the medication planning to facilitate implementation	Collective Action (Contextual Integration)
		Perceptions of the benefits and issues with using this approach to blood pressure management	Reflexive Monitoring (Individual appraisal)
	Using remote communication to manage blood pressure	Prescribers’ perceptions of implementing medication escalation remotely	Collective Action (Relational Integration, Interactional Workability)
		Supporters’ experiences of supporting patients via email	Collective Action (Relational Integration)
		Prescribers’ and supporters’ experiences of receiving emails from patients	Collective Action (Interactional workability)
	Delivering additional support to patients at the Practice	Perceptions about using the CARE approach to support patients	Coherence (Individual Specification) Collective Action (Skillset Workability)
Reluctance to escalate medication		Barriers to adhering to recommended medication escalations	Collective Action (Relational Integration)

**Table 5:** Adherence rates for target behaviours

Target behaviour	*N* incidents of adherence	Total possible incidents of adherence (*n*)	% adherence
Prescriber adherence to planning three medication escalations	231	283	81.63
Prescriber adherence to initiating recommended medication escalations within 28 days	215	405	53.09
Prescriber adherence to contacting patient remotely about a medication escalation	74	196	37.76
Supporter adherence to sending monthly support emails to patients	1611	2865	56.23

Most practitioners considered the intervention to be straightforward to implement and to fit well with normal practice (Table 4). The organisation of work between the prescriber and supporter was flexible, such that in some practices they worked very closely together and even shared some tasks, whilst in other practices they worked more independently.

In terms of implementing target behaviours, most prescribers created a three-step medication plan for their patients, but mean adherence rates for initiating medication escalations when recommended were moderate (53%). This was in agreement with the qualitative analysis, where some prescribers felt that escalating medication was straightforward, but a few were reluctant to make an escalation. This led to deviations in implementation as one prescriber-supporter, who implemented 4/24 recommended escalations during the trial (17%), preferred to check patients’ blood pressure readings in the clinic each time they were recommended a medication escalation. She believed that clinic readings were more reliable than using the average of seven home readings and suggested that home readings could be unreliable if, for example, the patient had not yet taken their medication that day.

“I normally do six readings myself here, just to make sure sort of it’s, you know, coinciding with their readings.. Sometimes when I've done it the readings have been quite different” (Prescriber-supporter 3).

Other prescribers described preferring to wait for more evidence from subsequent months of home monitoring before escalating medication and possibly trying lifestyle changes first.

Adherence to contacting patients remotely to notify them about a medication escalation was fairly low (38%), with telephone or face-to-face contact being more common. This was in line with mixed opinions about remote medication escalation in the process interviews where some prescribers felt changing medication remotely was efficient whilst others found it a hassle to amend the template letter or disliked having no record that it had been received, and so preferred to phone the patient.

“It’s easy, it’s quite nice because, you know, you don't need to contact the patient, you just do the prescription, print off that letter, and that’s quite nice, I like that.” (Prescriber 13)

There were also concerns that, although the three-step medication plan had been agreed with the patient in advance, the patient might want this information reiterating.

Adherence to sending monthly support emails to patients was moderate (56%), in agreement with the wide range of perceptions about using email to support patients. Supporters liked being provided with templates as this saved them time, and in some practices, the task was shared between staff or delegated to the administrative team. Having designated time helped supporters manage this task. However, it seemed that perceiving the process as straightforward was not sufficient to ensure high adherence; one supporter sent 27% of the monthly support emails despite describing the process as easy.

“I’ve just used your templates and that was fine. It’s quite easy to follow... I haven’t had any replies to my—I didn’t have any replies to my supportive emails” (Supporter 1)

The template emails were not designed to initiate spontaneous updates but many patients chose to reply to their supporters with updates. Two supporters with very high adherence rates (sending 95% and 118% of the planned emails respectively, including some ad hoc emails to patients) both described how their patients liked receiving the emails. Where supporters did not hear anything from their patients, they could feel frustrated that they were not more directly involved with patients’ blood pressure management.

“I've had nothing back, and nobody has asked to see me face to face…. …I suppose that really is a slight frustration, that you’re not getting much feedback from them. But I suppose, I would think that they feel because they’re in touch with the GP, they don’t really need to respond to me” (Supporter 11).

A minority of supporters felt that face-to-face support was more personal and easier for managing blood pressure. Two of these supporters still used the email system to some extent (20% and 42% adherence rates respectively), but the other chose to see all her patients in person and did not send any patient emails.

In terms of face-to-face support, most supporters had no experience of using the CARE approach due to low uptake of optional support appointments by patients. When prompted about using CARE to support patients, supporters perceived *Congratulation* and *Encouragement* to be in line with what they already do, although a couple felt reluctant to congratulate participants if their progress was limited, either because this could feel insincere or because they felt the patient had not made enough progress to warrant praise.

“It feels fake to congratulate. If there is not enough steps. Or if somebody says, “Oh I lost weight, half kilo.” Well, well done, but not excellent” (Supporter 7)

### Mechanisms of change

Table 6 shows that there was a statistically significant increase in scores on self-efficacy, outcome expectancies, and perceived acceptability of the intervention after training for both prescribers and supporters.

**Table 6:** Practitioner self-efficacy, outcome expectancies, and perceived acceptability questionnaire scores before and after training

Scale	Individual items where not treated as a scale	Response options	Before training median (range)	After training median (range)	Wilcoxon *z* score	95% CI for mean difference scores
Prescriber self-efficacy (*n* = 67)	a. Create individualised patient medication plans	1–10	9 (1–10)	10 (1–10)	− 5.20	0.59 to 1.30
	b. Increase patient medication when blood pressure remains too high		9 (1–10)	10 (1–10)	− 3.06	0.13 to 0.68
	c. Integrate the HOME BP programme in to regular care		7 (1–10)	9 (2–10)	− 5.95	1.41 to 2.38
Prescriber outcome expectancies mean score (*n* = 67)		1–5	4.00 (3–5)	4.17 (3.33–5.00)	− 5.09	0.19 to 0.36
Prescriber perceived acceptability of the intervention for patients (*n* = 67)	a. Self-monitor their blood pressure at home	1–10	7 (5–10)	8 (5–10)	− 4.96	0.62 to 1.30
	b. Enter their blood pressure readings in to HOME BP		7 (1–10)	8 (5–10)	− 4.72	0.80 to 1.65
	c. Make medication changes to control their blood pressure		6 (1–10)	8 (5–10)	− 5.57	1.23 to 2.28
Supporter self-efficacy mean score (*n* = 57)		1–10	7.67 (2.33–10)	9.33 (6.67–10)	− 5.55	1.32 to 2.33
Supporter outcome expectancies mean score (*n* = 57)		1–5	4.17 (3–5)	4.5 (3–5)	− 4.34	0.16 to 0.38
Supporter perceived acceptability of the intervention for patients mean score (*n* = 57)		1–10	6.67 (1–10)	8.33 (3.67–10)	− 4.82	0.88 to 2.00

Spearman’s correlations showed several significant relationships between self-efficacy items and prescriber adherence to initiating recommended medication escalations within the trial (Additional file 7). Relationships were found with scores both before and after prescribers completed the online intervention training. Also in line with the logic model, prescribers who adhered to planning medication escalations were more likely to escalate medication when recommended (*r* = .29, *p* < .05). Outcome expectancies and perceived acceptability of the intervention for patients were not associated with adherence to any prescriber target behaviours, and no relationships were found for supporters between their questionnaire scores pre- or post-training and their adherence to sending monthly support emails.

The qualitative data suggested there may also be other mechanisms influencing practitioners’ adherence to medication escalation. Some prescribers believed in the necessity of escalating medication at the thresholds used in this RCT, with one suggesting that the notifications needed to be more directive to leave less room for inaction, and a prescriber-supporter describing how she overcame reluctance from her patients to escalate medication.

“I think there's a lot of them make excuses, so “I drink a lot of caffeine” and this kind of thing… And I just say to them “Well, it’s been a couple of months now and it’s high and I think we just need to start new medication” (Prescriber-supporter 2)

However, others decided against medication escalation due to low perceived necessity, or concerns about patients’ blood pressure going too low.

“The research GP… said, “Look”—after discussion with patient of course—“I’m not happy to escalate it. If I escalate your dose you will go into hypotension, you will be faint-y, you will be dizzy. It’s just—shall we try perhaps next month?”. (Supporter 7).

### Context

The mixed-methods triangulation found that despite high adherence to planning medication changes, several contextual barriers were raised to implementing this process in the qualitative interviews.

These included when the patient was already taking multiple medications or had a history of side effects, which ruled many potential medications out. The patient experiencing side effects during the RCT also made the three-step plan less feasible to implement, as practitioners then had to revise the plan which led to concerns about patient anxiety, or frustration at the additional work.

“You’ve got a plan and now that’s changing and now do I have to make another three-point plan? And that’s really irritating and now I’ve gone off piste” (Prescriber 1)

Contextual factors also influenced practitioners’ decisions about whether to escalate medication when recommended. Recommendations based on higher systolic readings were more likely to be adhered to (*d* = 0.41), see Additional file 7. This was in line with the qualitative analysis in which a practitioner who adhered to 0/2 medication escalation recommendations described how the proximity of the patient’s average to the threshold led him to call the patient to discuss the medication escalation, and they jointly agreed not to escalate the dose.

“The recommendations were to up the medication even though they were only one systolic point, on average, over, over the target, and that sort of, you know the patient was very reluctant to change that, so we agreed that we wouldn’t proceed to that next step” (Prescriber 5)

Recommendations for medication escalation later in the RCT and when a higher number of recommendations for medication escalation had already been made for a patient were also less adhered to (accounting for 7% and 8% of the variance respectively); see Additional file 7.

Table 7 shows the triangulation of key qualitative and quantitative findings.

**Table 7:** Triangulation outcomes from integrating quantitative and qualitative data

Quantitative data finding	Qualitative data finding	Triangulation outcome
Prescribers’ and supporters’ post-training questionnaires showed positive outcome expectancies and high confidence in intervention acceptability.	Practitioners perceived the digital intervention as a more accurate way of managing blood pressure and as being in line with the direction of Primary Care.	Partial agreement (complementary findings)
No quantitative data were collected on setting up and integrating the digital intervention in normal practice.	Most practitioners considered that the programme was easy to integrate and described flexible approaches to organising the work.	Silence
Adherence to planning three medication escalations was high (82%). Social cognitive beliefs and perceived acceptability of the intervention were not associated with adherence to planning medication escalations.	Whilst some prescribers perceived planning medication facilitated more comprehensive care, others described issues with planning in advance, including patient anxiety and additional effort when the plan needed revising.	Dissonance
Adherence to initiating medication escalations was moderate (53%). Pre-planning medication escalations, self-efficacy beliefs and contextual patient factors such as average blood pressure reading and *n* of previous recommendations were related to adherence to initiating medication escalation.	Some prescribers believed that changing medication in response to recommendations was straightforward, but some reasons were discussed for not changing medication, including readings being close to the threshold, concerns about hypotension, and preferring to wait for more evidence.	Agreement
Adherence to remotely changing medication was fairly low (38%).	Prescribers described preferring real-time contact at the time of a medication escalation in order to ensure patients have understood, and to avoid the hassle of sending a letter.	Agreement
Adherence to sending patient support emails was moderate (56%). Social cognitive beliefs and perceived acceptability of the intervention were not associated with adherence to sending patient support emails.	Perceptions about supporting patients by email were mixed. Positive feedback from patients about the emails seemed to promote the perceived value of email support for supporters.	Agreement
No quantitative adherence data were collected on using the CARE approach.	Supporters described a very low uptake to appointments by patients, so many had no experience of using CARE in practice. Hypothetical concerns included how to congratulate when patients’ progress was limited, and how to avoid giving advice when the patient expected it.	Silence

## Discussion

This mixed-methods process evaluation revealed that a digital intervention to overcome clinical inertia for hypertension was implemented with moderate adherence by healthcare practitioners.

In terms of mechanisms, the logic model was partially supported in that self-efficacy was associated with adherence to escalating medication when recommended, but outcome expectancies were not. Thematic analysis provided insights into additional pathways which might influence implementation, such as individual practitioner beliefs about the necessity to escalate medication when readings were close to the target threshold, and concerns about risks of hypotension (low blood pressure), supporting the Necessity-Concerns framework [34].

In terms of context, patients’ average reading and the number of previous recommendations to escalate medication influenced adherence to medication escalation, in line with previous research [26, 35, 36]. This suggested an issue with sustainability of the intervention, with lower adherence to recommendations made later on in the trial when prescribers might have already tried a medication escalation which had been ineffective.

### Distinguishing non-adherence from appropriate adaptation

A challenge for process evaluations is distinguishing between innovative adaptations to account for contextual variation and subversion or infidelity to intervention procedures [14]. In this trial, practitioners initiated medication escalation in response to 53% of recommendations, which is comparable to a previous hypertension tele-monitoring trial in which medication escalations were patient-initiated (55%) [12], and exceeds a US tele-monitoring trial in which physicians initiated 41% of recommended changes [31]. However, despite only moderate adherence to medication escalations, the RCT found that HOME BP did significantly reduce blood pressure in the intervention group [12]. Therefore, is moderate adherence to medication escalation sufficient, or even optimal, and could cases of non-adherence be described as innovation rather than subversion?

An expert consensus study produced a six-point checklist defining circumstances in which not escalating medication for uncontrolled hypertension in Primary Care could be deemed appropriate inaction, specifically: when raised BP has not been confirmed by home readings; legitimate doubt exists about the reliability of the readings; suspected patient non-adherence to medication; specific patient characteristics increase risk of hypotension;a more urgent medical priority takes precedence; or there is difficulty accessing treatment [37]. Of the reasons influencing implementation of medication escalation in this process evaluation, concerns about risk of hypotension would fit these criteria for appropriate inaction, although no guidance was provided around the patient characteristics which warrant such concerns. Low perceived necessity due to proximity of readings to the threshold, and perceiving the average of home readings to be generally unreliable, would be classified more as clinical inertia, as home readings are recommended by NICE as an effective indicator to manage blood pressure [38]. This suggests that strategies to address these barriers to implementation may enhance intervention effectiveness.

This study also identified adaptations to the use of one-way notifications by letter or email to notify patients about medication escalations or offer ongoing support. These processes were adapted in unanticipated ways to facilitate two-way communication, such as supporters providing patients with their personal email addresses, and patients responding to support emails via the intervention, suggesting a preference on both sides for more interaction. The qualitative interviews indicated that practitioners felt uncertain about whether remote support could meet patients’ needs, especially when they received no response, which is consistent with evidence that practitioners believe in-person support to be higher quality and more in line with their role [23, 39, 40].

### Implications for future research

The findings were mapped on to NPT to help identify how these barriers influenced implementation, and possible strategies are suggested with reference to the Expert Recommendations for Implementing Change (ERIC) taxonomy [41]. These are shown in Table 8 and could help inform future research in clinical inertia and digital interventions. Stakeholder involvement or co-production with practitioners could be used to explore how these potential strategies could be most feasibly implemented to address the complexities of clinical inertia [43].

**Table 8:** Barriers to implementation of target behaviours mapped onto NPT mechanisms, and possible solutions mapped onto the Expert Recommendations for Implementing Change taxonomy

Barrier to implementation	NPT mechanism	Possible solution	Expert Recommendations for Implementing Change (ERIC) taxonomy
Doubts about the thresholds used to escalate medication	Low coherence	Adjusting the mismatch between the legislative targets of 150/90 mmHg (NHS England 2018) and the evidence-based targets of 135/85mmHg.	Involve executive boards
For some practitioners, applying an algorithm to promote clinical decisions creates perceived conflict with delivering patient-centred care and shared-decision making	Low cognitive participation	Using an approved checklist [37] to inform criteria for distinguishing appropriate inaction from clinical inertia, to allow clinicians more flexibility in decision-making, whilst still encouraging medication escalation in cases where clinical inertia can occur. Where a practitioner decides not to escalate medication, the checklist could prompt them to plan when they will review their decision and any interim actions agreed with the patient, such as lifestyle change.	Promote adaptability
Patients’ blood pressure readings are close to the target	Low coherence	Tailored email prompts with evidence for the benefits and safety of lowering blood pressure below the target.	Tailor strategies
Wanting to wait for more evidence from further home blood pressure readings before making a medication change	Low interactional workability	Improved tracking capacity to allow practitioners to view patients’ readings over time and see cumulative evidence for medication escalation. Clinical Performance Feedback Intervention Theory describes several mechanisms for optimising the effectiveness of audit and feedback systems, including trends to show patient’s performance over time, and benchmarking to allow comparison with other practitioners [42].	Audit and provide feedback
Concerns about risk of hypotension following a medication change	Low reflexive monitoring	Tracking could reduce perceived risk of escalating medication by enabling practitioners to check patients’ clinical status after an escalation.	Audit and provide feedback
GPs’ concerns about one-way notifications for patients not being received	Low cognitive participation	Some SMS systems already used in Primary Care allow patients to rapidly acknowledge receipt, which could increase feasibility of patient notifications for GPs.	Obtain and use feedback from patients/consumers and family
Some nurses had concerns that one-way notifications conflict with their role of providing tailored patient support	Low coherence	Provide facility to allow nurses to enable two-way communication with patients if they wish to.	Involve patients/consumers and family members

### Implications for implementation science

Working closely with practitioners during the design of a digital intervention is essential both for overcoming any perceived conflict between the digital intervention procedures and practitioners’ perceived role and selecting sensitive quantitative measures to evaluate mechanisms during process evaluation. For HOME BP, in-depth focus groups with practitioners were conducted during intervention development which informed important optimisations to the intervention training [6], but ethnographic observations of practitioners conducting intervention procedures with patients could further enhance understanding of how to ensure intervention processes are perceived as compatible with practitioners’ role and how to support practitioners to bridge the gap where one is perceived. Such ethnographic observations could also have helped highlight the value of capturing additional mediating mechanisms which appeared to be important influences on practitioners’ implementation of the intervention, specifically perceived necessity and risk.

Future process evaluations could also consider a longitudinal approach to exploring changes in perceptions of implementation with the same practitioners over time. This would enable clearer insights into how a new process is adopted and monitored over time, with each experience of the intervention influencing practitioners’ *Reflective monitoring* and ongoing engagement [44].

### Strengths and limitations

This detailed mixed-methods process evaluation has enabled a more nuanced understanding of the implementation of a digital intervention in Primary Care, helping to build knowledge of determinants of implementation and inform the selection of possible strategies, in line with current guidance [43].The rigour and coherence of the interpretations were supported by their consistency with the literature, theory, and with each other [45].

Additional methods, such as recordings of consultations to explore how practitioners and patients interact when planning or escalating medication, or questionnaires to explore beliefs about medication escalation and contextual variations between sites might further enhance understanding of the barriers to these key behaviours.

It should be noted that whilst the gender distribution of supporters in the trial (95% female) was approximately consistent with that of nurses or healthcare assistants in Primary Care (97% female), only 35% of prescribers were female compared with 57% of General Practitioners across the UK [46]. This finding could influence the generalisability of the findings as gender has been shown to influence clinical decision making, with female clinicians spending more time on disease prevention [47]. The sample was too small to allow sub-group comparisons of adherence to medication escalation by gender, but this limitation should be considered when evaluating the intervention’s transferability to UK Primary Care.

## Conclusions

This mixed-methods process evaluation showed that a digital intervention to address clinical inertia in hypertension was implemented with moderate adherence, with diverse perceptions of implementation amongst practitioners across 70 GP Practices. Implementation was associated with practitioners’ self-efficacy to use intervention procedures, although beliefs about perceived necessity of escalating medication and concerns about patient risk also appeared important mechanisms. Contextual factors influencing adherence to medication escalation included proximity of patients’ average reading to target thresholds, and the number of previous recommendations made to escalate a patient’s medication, such that adherence reduced over time. NPT helped understand the mismatch between high practitioner self-efficacy and moderate adherence, showing that low *Coherence* of the intervention could impede incorporation of these new procedures into practice. Implementation strategies to improve feasibility of interventions to address clinical inertia could include promoting adaptability and tailoring strategies.

Digital interventions should also consider whether target behaviours are in line with practitioners’ values. Patient notifications may be more feasible to implement if clinicians receive acknowledgement from patients that they have received the information, whilst nurses may be more willing to use email when patients can send responses, enabling personalised support. Such additional features would need to be evaluated to ensure they do not increase burden on practitioners.

## Supplementary Information

**Additional file 1.**

**Additional file 2.**

**Additional file 3.**

**Additional file 4.**

**Additional file 5.**

**Additional file 6.**

**Additional file 7.**

## Data Availability

The qualitative transcripts generated and/or analysed during the current study are not publicly available due to protecting participants’ anonymity. The quantitative datasets are available from the corresponding author on reasonable request.

## Abbreviations

AMUSED: Analysing and Measuring Usage and Engagement Data
CARE: Congratulate, Ask, Reassure, Encourage
GP: General Practitioner
GRAMMS: Good Reporting of A Mixed Methods Study
IMD: Index of Multiple Deprivation
NPT: Normalisation Process Theory
RCT: Randomised controlled trial
SCT: Social Cognitive Theory
STaRI: Standards for Reporting Implementation Studies
