# Supplementary material for: Implementing a digital intervention for managing uncontrolled hypertension in Primary Care: a mixed methods process evaluation
Source: Implement Sci. 2021 May 26;16:57. doi: 10.1186/s13012-021-01123-1 (PMC8152066; doi:10.1186/s13012-021-01123-1)
Supplement: Supplementary file 1 — Additional file 1. [file 13012_2021_1123_MOESM1_ESM.docx]

**Additional file 1. StaRI checklist and GRAMMS checklist**

STaRI checklist: Standards for Reporting Implementation Studies

| **Checklist item** | | **Reported on page #** | **Implementation Strategy** | | | **Reported on page #** | **Intervention** |
| --- | --- | --- | --- | --- | --- | --- | --- |
|  | |  | “Implementation strategy” refers to how the intervention was implemented. | | |  | “Intervention” refers to the healthcare or public health intervention that is being implemented. |
| **Title and abstract** | | | | | | | |
| Title | **1** | 1 (title) | Identification as an implementation study, and description of the methodology in the title and/or keywords | | | | |
| Abstract | **2** | 3 (abstract) | Identification as an implementation study, including a description of the implementation strategy to be tested, the evidence-based intervention being implemented, and defining the key implementation and health outcomes. | | | | |
| **Introduction** | | | | | | | |
| Introduction | **3** | 6 (background) | | Description of the problem, challenge or deficiency in healthcare or public health that the intervention being implemented aims to address. | | | |
| Rationale | **4** | 6 (background para 1 & 2) | | The scientific background and rationale for the implementation strategy (including any underpinning theory/framework/model, how it is expected to achieve its effects and any pilot work). | | 8-10  (Figure 2) | The scientific background and rationale for the intervention being implemented (including evidence about its effectiveness and how it is expected to achieve its effects). |
| Aims and objectives | **5** | 7  (background) | | The aims of the study, differentiating between implementation objectives and any intervention objectives. | | | |
| **Methods: description** | | | | | | | |
| Design | **6** | 7-8 (design) | | | The design and key features of the evaluation, (cross referencing to any appropriate methodology reporting standards) and any changes to study protocol, with reasons | | |
| Context | **7** | 8 (intervention and proposed mechanisms of action) | | | The context in which the intervention was implemented. (Consider social, economic, policy, healthcare, organisational barriers and facilitators that might influence implementation elsewhere). | | |
| Targeted ‘sites’ | **8** | 12 (participants) | | | The characteristics of the targeted ‘site(s)’ (e.g locations/personnel/resources etc.) for implementation and any eligibility criteria. | Reported elsewhere | The population targeted by the intervention and any eligibility criteria. |
| Description | **9** | 7-8 (design)  11 (Table 2) | | | A description of the implementation strategy | 8-10  (Table 1 and Figure 2) | A description of the intervention |
| Sub-groups | **10** | 12 (participants) | | | Any sub-groups recruited for additional research tasks, and/or nested studies are described | | |
| **Methods: evaluation** | | | | | | | |
| Outcomes | **11** | 12-13 (analysis) | | | Defined pre-specified primary and other outcome(s) of the implementation strategy, and how they were assessed. Document any pre-determined targets | 11  (Table 2) | Defined pre-specified primary and other outcome(s) of the intervention (if assessed), and how they were assessed. Document any pre-determined targets |
| Process evaluation | **12** | 7 (background)  10 (figure 2) | | | Process evaluation objectives and outcomes related to the mechanism by which the strategy is expected to work | | |
| Economic evaluation | **13** | N/A | | | Methods for resource use, costs, economic outcomes and analysis for the implementation strategy | N/A | Methods for resource use, costs, economic outcomes and analysis for the intervention |
| Sample size | **14** | 12 (participants) | | | Rationale for sample sizes (including sample size calculations, budgetary constraints, practical considerations, data saturation, as appropriate) | | |
| Analysis | **15** | 12-14  (analysis and integration) | | | Methods of analysis (with reasons for that choice) | | |
| Sub-group analyses | **16** | N/A | | | Any a priori sub-group analyses (e.g. between different sites in a multicentre study, different clinical or demographic populations), and sub-groups recruited to specific nested research tasks | | |
| **Results** | | | | | | | |
| Characteristics | **17** | 15 (Table 3 ) | Proportion recruited and characteristics of the recipient population for the implementation strategy | | | Reported elsewhere | Proportion recruited and characteristics (if appropriate) of the recipient population for the intervention |
| Outcomes | **18** | 15-16 (results) and Additional file 7 | Primary and other outcome(s) of the implementation strategy | | | Reported elsewhere | Primary and other outcome(s) of the Intervention (if assessed) |
| Process outcomes | **19** | 18-19 | Process data related to the implementation strategy mapped to the mechanism by which the strategy is expected to work | | | | |
| Economic evaluation | **20** | N/A | Resource use, costs, economic outcomes and analysis for the implementation strategy | | | N/A | Resource use, costs, economic outcomes and analysis for the intervention |
| Sub-group analyses | **21** | N/A | Representativeness and outcomes of subgroups including those recruited to specific research tasks | | | | |
| Fidelity/ adaptation | **22** | 14-18  (results) | Fidelity to implementation strategy as planned and adaptation to suit context and preferences | | | 13-23 (results) | Fidelity to delivering the core components of intervention (where measured) |
| Contextual changes | **23** | 19-20 (results) | Contextual changes (if any) which may have affected outcomes | | | | |
| Harms | **24** | 14-23 (results and discussion) | All important harms or unintended effects in each group | | | | |
| **Discussion** | | | | | | | |
| Structured discussion | **25** | 20-24 (discussion) | Summary of findings, strengths and limitations, comparisons with other studies, conclusions and implications | | | | |
| Implications | **26** | 23 (discussion) | Discussion of policy, practice and/or research implications of the implementation strategy (specifically including scalability) | | | 22 (Table 8) | Discussion of policy, practice and/or research implications of the intervention (specifically including sustainability) |
| **General** | | | | | | | |
| Statements | **27** | 4, 8, 27 | Include statement(s) on regulatory approvals (including, as appropriate, ethical approval, confidential use of routine data, governance approval), trial/study registration (availability of protocol), funding and conflicts of interest | | | | |

GRAMMS checklist: Good Reporting of A Mixed Methods Study

| **Guideline** | **Section: page** |
| --- | --- |
| Describe the justification for using a mixed methods approach to the research question | Background: p6 |
| Describe the design in terms of the purpose, priority and sequence of methods | Design: p7-8 |
| Describe each method in terms of sampling, data collection and analysis | Data collection: p10-11  Participants: p12  Analysis: p12-14 |
| Describe where integration has occurred, how it has occurred and who has participated in it | Design: p7-8  Integration: p14 |
| Describe any limitation of one method associated with the present of the other method | Strengths and limitations: p23-24 |
| Describe any insights gained from mixing or integrating methods | Results: p14-20 |
