# Supplementary material for: Implementing a digital intervention for managing uncontrolled hypertension in Primary Care: a mixed methods process evaluation
Source: Implement Sci. 2021 May 26;16:57. doi: 10.1186/s13012-021-01123-1 (PMC8152066; doi:10.1186/s13012-021-01123-1)
Supplement: Supplementary file 2 — Additional file 2. [file 13012_2021_1123_MOESM2_ESM.docx]

**Additional file 3. Example of a template email provided for supporters to send to patients.**

Dear *(insert patient's name),*

I hope that this email finds you well. I was just wondering how you are getting on with monitoring your BP, I hope this is going well.

I've been reading about all the benefits that eating a healthy diet and being more active can bring you. These habits are great for bringing down your BP, but also really fantastic for preventing cancer, dementia and heart disease! I wonder how you are getting on with your healthy habits.

Often people find that they slip up from time to time, the best way to start again is just to draw a line under it and decide that today is a new day- a new opportunity to do something positive for yourself.

(*If you think your patient isn’t doing a healthy habit then omit this highlighted section)*

Remember, if you get stuck or have any problems you can always send me a message through HOME BP- I'll always do my best to help.

Take care,

(insert name)

HOME BP (insert title and practice)
