# Supplementary material for: Implementing a digital intervention for managing uncontrolled hypertension in Primary Care: a mixed methods process evaluation
Source: Implement Sci. 2021 May 26;16:57. doi: 10.1186/s13012-021-01123-1 (PMC8152066; doi:10.1186/s13012-021-01123-1)
Supplement: Supplementary file 3 — Additional file 3. [file 13012_2021_1123_MOESM3_ESM.docx]

**Additional file 3. Automated email prompts received by Practitioners to escalate patients’ medication**

Email sent to prescriber following two consecutive months of raised average readings:

*Email subject line: Prescriber ACTION REQUIRED - HOME BP patient requires a medication change*

Dear HOME BP Prescriber,

Your patient <insert username> has had BP readings that have remained above target for two consecutive months and is now required to make a medication change. They have been informed of this. Remember that the study protocol is for medication changes to go ahead without seeing the patient, unless there are unusual clinical circumstances that mean you need to see them. Specifically this email will provide you with the HOME BP data in order to facilitate titration without increasing workload in clinic. Most patients are happy to start or adjust a new medication change without seeing their GP.

Their readings last time they monitored were: <insert readings > The patient’s new readings from the last week are: <insert new readings>.

Remember that the target thresholds for the study are based on the NICE guidelines for home BP readings: 135/85 for patients under 80 without diabetes, 135/75 for patients with diabetes, and 145/85 for patients over 80 without diabetes. The medication titration procedure is outlined below:

- 1. Print the prescription for the next medication change (you previously saved a list of future planned medication changes in the patient’s notes).
  2. Next complete the ‘Patient medication letter’ which your practice manager has saved on your computer system – you can also access a copy of this letter in HOME BP, just click the following link and go to the Forms area of the menu <insert link>.
  3. Now post the prescription and patient medication letter to your patient, or alternatively leave these with the reception staff and ask reception to call the patient to collect their prescription and letter.
  4. Save the letter to the patient notes, or use any other method that you wish to indicate in the notes that you have implemented the medication change.

For further information about the titration procedures or any other study information just click on the link <insert link >

If this link doesn’t work for any reason, then you can open a new web page and copy and paste the link into the web-address bar at the top of your new web page. If you have any problems then you can also contact the study team (details below).

Thanks again for your help with this study.

From the HOME BP Team

Email sent to prescriber following a one-off very high or very low reading:

*Email subject line: Prescriber ACTION MAY BE REQUIRED - Patient has had a red/blue reading*

Dear HOME BP Prescriber,

Your patient <insert username> has just entered their BP readings into HOME BP and they had a red reading, which means their BP was too high. When receiving their first red reading, the patient was asked to take their BP again twice more, each after 5 minutes of rest. The reading they entered should therefore be their third consecutive red reading.

Their readings from their week of home monitoring were: <insert BP readings>.

The patient may have already contacted you about this, if not then please get in touch with them as soon as possible to discuss this red reading and check whether they need a medication change. Remember that the study protocol is for medication changes to go ahead without seeing the patient, unless there are unusual clinical circumstances that mean you need to see them. Specifically this email provides you with the HOME BP data in order to facilitate titration without increasing workload in clinic. Most patients are happy to start or adjust a new medication change without seeing their GP.

For further information about the titration procedures or any other study information just click on the link <insert link > If this link doesn’t work for any reason, then you can open a new web page and copy and paste the link into the web-address bar at the top of your new web page. If you have any problems then you can also contact the study team (details below).

Thanks again for your help with this study.

From the HOME BP Team
