# Supplementary material for: Implementing a digital intervention for managing uncontrolled hypertension in Primary Care: a mixed methods process evaluation
Source: Implement Sci. 2021 May 26;16:57. doi: 10.1186/s13012-021-01123-1 (PMC8152066; doi:10.1186/s13012-021-01123-1)
Supplement: Supplementary file 4 — Additional file 4. [file 13012_2021_1123_MOESM4_ESM.docx]

**Additional file 4. Practitioner questionnaire completed before and after online training at baseline**

The self-reported questionnaires were answered using Likert Scales to indicate agreement, and were designed using social-cognitive theory (SCT). SCT constructs were identified as proposed theoretical mediators in the HOME BP logic model (12) (Figure 1).

Prescribers

These questions are designed to get a better understanding of different aspects of hypertension management.

Please rate how sure you are that **you** could do these behaviours right now by using the sliding scales given below: *(not at all sure – very sure)*

1. Create individualised patient medication plans
2. Increase patient medication when blood pressure remains too high
3. Integrate the HOME BP programme in to regular care

Please rate how sure you are that **your patients** could do these behaviours right now by using the sliding scales given below: *(not at all sure – very sure)*

1. Self-monitor their blood pressure at home
2. Enter their blood pressure readings in to the HOME BP programme
3. Make medication changes to control their blood pressure

Please rate how strongly you agree with each of the following statements:

(1 = strongly disagree, 2 = disagree, 3 = neutral, 4 = agree, and 5 = strongly agree)

1. Blood pressure monitoring will improve patient hypertension self-management
2. The HOME BP programme will improve patient hypertension self-management
3. Pre-planned medication changes will improve patient hypertension self-management
4. Blood pressure self-monitoring will improve patient blood pressure control
5. The HOME BP programme will improve patient blood pressure control
6. Pre-planned medication changes will improve patient blood pressure control

Supporters

These questions are designed to get a better understanding of different aspects of hypertension management.

Please rate how sure you are that **you** could do these behaviours right now by using the sliding scales given below: *(not at all sure – very sure)*

1. Support patients to self-monitor their blood pressure
2. Use the HOME BP programme to support patients
3. Integrate the HOME BP programme in to regular care

Please rate how sure you are that **your patients** could do these behaviours right now by using the sliding scales given below: *(not at all sure – very sure)*

1. Self-monitor their blood pressure at home
2. Enter their blood pressure readings in to the HOME BP programme
3. Make medication changes to control their blood pressure

Rate how strongly you agree with each of the following statements:

(1 = strongly disagree, 2 = disagree, 3 = neutral, 4 = agree, and 5 = strongly agree)

1. BP monitoring will improve patient hypertension self-management
2. The HOME BP programme will improve patient self-management
3. Pre-planned medication changes will improve patient hypertension self-management
4. BP self-monitoring will improve patient blood pressure control
5. The HOME BP programme will improve patient blood pressure control
6. Pre-planned medication changes will improve patient blood pressure control
