# Supplementary material for: Implementing a digital intervention for managing uncontrolled hypertension in Primary Care: a mixed methods process evaluation
Source: Implement Sci. 2021 May 26;16:57. doi: 10.1186/s13012-021-01123-1 (PMC8152066; doi:10.1186/s13012-021-01123-1)
Supplement: Supplementary file 5 — Additional file 5. [file 13012_2021_1123_MOESM5_ESM.docx]

**Additional file 5. Practitioner process interview schedule**

**General opening question(s)**

1. I’m really interested in hearing how you have got on with the HOME BP study so far, please can you tell me all about how you are finding it?
2. What did you think of the prescribers’ guide?
3. How easy has it been to follow the guidance given in the prescribers’ guide?
4. Overall, what have you liked about the HOME BP programme so far?
5. What have you disliked about the HOME BP programme so far?
6. How would you like to see the HOME BP programme improved?

**More specific questions:**

1. How have you found the baseline medication review with patients in usual care?
2. How have you found the baseline medication review with patients in the intervention group?
3. What worked well?
4. What worked less well?

If haven’t discussed: How are you finding it deciding patients’ potential medication changes in advance?

**Now thinking about those in the intervention group:**

With respect to changing a patient’s medications while they were in the study:

1. How have you found the medication change process?
2. After you saw the email about the medication change, what happened next?
   1. Did you see a patient when the medication change was due?
   2. If yes – How did you come to meet with the patient?
   3. Can you tell me more about this appointment? (Aiming to find out who initiated it – patient or GP)
3. Have you had any patients who have had a red or blue reading?
   1. If yes, how did you find the process of dealing with this?
4. How has it been for you receiving emails from the HOME BP programme?

**Questions about supporting:**

1. How do you think patients are finding the study so far?
2. How have you found it acting as both the prescriber and the supporter for the study?
3. Overall, how do you feel about supporting patients who are using HOME BP?
4. Thinking about patients’ screening appointments where you took consent and baseline clinical measures – how did you find these?
   1. What did you find worked well?
   2. And what worked less well?

Prompt (if not covered in replies): Why do you think this is?

If they raise problems: Can you think of any solutions?

1. Thinking about the first optional support session at around 4 weeks after a patient is randomised, have any of your patients chosen to come in for an appointment with you about monitoring their blood pressure?

**If yes:** how have you found these appointments?

1. What are you finding works well in this appointment?
2. And what works less well?

If they raise problems: Can you think of any solutions?

**If no:** How would you feel about having one of these appointments if a patient did want to see you at this point?

1. Patients in the intervention group are asked to practice monitoring their blood pressure for one week. Did any patients email you their practice blood pressure readings?

If so, how did you find this process?

1. Thinking about the second optional appointment at around 10 weeks after a patient is randomised, have any of your patients chosen to come in for an appointment with you to talk about choosing a lifestyle change that they want to make?

If yes: how are you finding this?

1. What are you finding works well in this appointment?
2. And what works less well?

If any problems: Can you think of any solutions?

1. How have you found using the CARE approach? (Congratulate, Ask, Reassure, Encourage)
2. What do you like about it?
3. What do you dislike about it?
4. If the supporter raises that they didn’t use the approach or did something else instead then ask them ‘Could you tell me about what you did in the support appointments with the patients.’
5. How easy has it been so far to follow the guidance given in the supporters’ guide?
6. How has it been giving patient support either face to face, by email or by phone?
7. What sort of things have you liked about these methods?
8. What sort of things have you disliked about these methods?
9. Why do you think that might be?
10. Can you think of any solutions?
11. How has it been sending a support email to each patient once a month through the HOME BP programme?
12. Have you received any emails from patients? How has it been responding to these emails?

Is there anything else that you would like to tell me about the HOME BP programme that we haven’t already discussed?

Do you have any questions at all before we finish?
