# Supplementary material for: Implementing a digital intervention for managing uncontrolled hypertension in Primary Care: a mixed methods process evaluation
Source: Implement Sci. 2021 May 26;16:57. doi: 10.1186/s13012-021-01123-1 (PMC8152066; doi:10.1186/s13012-021-01123-1)
Supplement: Supplementary file 6 — Additional file 6. [file 13012_2021_1123_MOESM6_ESM.docx]

Supplementary file 1: Coding manual for HCP process interviews

| **Theme** | **Sub-theme** | **Code** | **Definition** | **Quote** |
| --- | --- | --- | --- | --- |
| **Ease or burden of implementing HOME BP** |  | Fits with what I already do | Considering on a practical level whether the study procedures were feasible. Includes comparison with current role or how easily the tasks fitted in with what they already do. | “I think I’ve liked its simplicity and the way it fits in quite well into, sort of, normal work really” (Prescriber 2) |
|  |  | Clear about my role and what to do at each point | Learning and understanding what was involved in their role | “The website is a fountain of knowledge. So if I've any uncertainty, I really like having somewhere else to go electronically, rather than going through the site files” (Supporter 8) |
|  |  | Managing HOME BP emails | Includes discussion of the number of emails from HOME BP, or how these were dealt with. | “We couldn’t quite work out how to make sure that emails weren’t missed if somebody was off sick or on holiday. So we had them sent to the generic email address as well, which meant I always received it twice, which can cause some confusion as to whether I’ve actioned it or not” (Supporter 6) |
|  |  | Prescriber and supporter roles complement each other | Working with the prescriber/ supporter during the study and how this worked in practice | “When I receive the, you know, the escalation dose email I always contact the GP because they know – they work part time as well, so might not have necessarily picked up the email” (Supporter 7) |
| **Belief in the concept of HOME BP** |  | Fit with direction of Primary Care management of blood pressure | General buy-in to the idea of the study and what it was trying to achieve. | “a more efficient, effective way of managing people with blood pressure, with high blood pressure. And, you know, minimising appointment time, and sort of, again, maximising patient, sort of, feeling of self-control” (Prescriber 2) |
|  |  | Promotes patient empowerment and compliance | Discussing advantages for patients of engaging in self-management, such as empowerment or improved compliance | “I like that it empowers the patients to self-manage. And, again, I think that that’s important in lots of ways. Particularly in something like blood pressure where patients don’t feel ill, so you’re encouraging them to take a medication and they can’t see why. So with Home BP they can see what’s happening with their blood pressure, and they can see what their medication’s doing” (Prescriber-supporter 1) |
|  |  | Regular, more accurate approach to monitoring BP readings | Comparisons of home readings to clinic readings for informing BP management. | “I’m sure that’s more accurate than the kind of ad hoc way we do it at the moment with a mixture of home readings and surgery readings” (Prescriber 10) |
| **Supporting patients to manage their own blood pressure** | Planning medication escalations | Planning changes was straightforward or tricky | How easy was it to plan medication changes in advance? | "It depends on their previous experience and what they’ve already had. Because sometimes you do find yourself slightly boxed into a corner about what medication choices you can make, and trying to come up with one medication change can sometimes be tricky" (Prescriber 3) |
|  |  | Adaptions to medication planning to facilitate implementation | Procedures outside study training which prescriber uses at baseline review | “The sheet of what they should do, the steps, that thing. I photocopy and fill it in for the patient so they know what steps they need to do if… if they need to make a change” (Prescriber 2) |
|  |  | Developing a more comprehensive care plan | Benefits of planning medication changes in advance in terms of having a holistic approach to BP management. | “We were already thinking two steps ahead in terms of medication changes. Which is nice to think about and nice to have a plan for” (Prescriber 9) |
|  |  | Disconcerted by deciding medication escalations in advance | Any concerns about planning medication in advance in terms of risk. | “Coming up with three forward interventions when you’ve not even tried one. And – you know, normally you get patients back after the first change of medication and see how they’re getting on with side effects, and whether they’re willing to carry on, and whether they need to change again, and there’s a bit of two-way conversation” (Prescriber 10) |
|  |  | That’s the whole problem with plans, when they go wrong | Issues with the 3-step medication plan needing changing | “I think then you’re worried about – that you’ve got a… it sounds a bit—it sounds stupid, but, you know, you’ve got a plan and now that’s changing and now do I have to make another three-point plan? And that’s really irritating” (Prescriber 1) |
|  | Using remote communication to manage blood pressure | Initiating medication escalation remotely | How did escalating patients’ medication remotely work in the study? | “I left the prescription with reception and told them to phone him, to tell him that his prescription's ready to pick up. That worked well, actually. That did work well. And, you know, he was really engaged and knew exactly what was going on and he was obviously confident to just, you know, if it was high, take medication as we discussed” (Prescriber 12) |
|  |  | Supporters’ experiences of supporting patients via email | The procedure for sending monthly emails and perceptions about the email content. | “They are ready-made templates. That's very useful because if I have to sort of come up with an email, that would be very, very time-consuming” (Supporter 10) |
|  |  | Perceptions of additional patient contact via email | When patient sends additional information by email, how is this interpreted? | “It provides another avenue for a patient to get hold of you…although we say to patients, “All right, tell us, you know, just email us your readings,” they email readings plus a whole narrative of what's going on in their life” (Prescriber 13)  “Some of them have actually sent me separate emails just to say, “Oh my blood pressure was a bit high on such a such a day, because I was stressful at work,” or something. So that was quite helpful.” (Supporter 8) |
|  | Delivering additional support to patients at the Practice | Using the CARE approach | Supporters’ feelings about using the CARE approach | “Your instructions are quite prescriptive, aren’t they, really? That-that you… As I understand it, you can’t really say a lot, only encourage them to-to do what they’ve been told on the… on the website”. (Prescriber-supporter 1)  “Situations for example where it might be overdone, a patient is on the study, let's say two months and I don't know, the BP is going slowly down but very slowly. It feels fake to congratulate. If there is not enough steps. Or if somebody says, “Oh I lost weight, half kilo.” Well, well done, but not excellent” (Supporter 7). |
| **Reluctance to escalate medication** |  | Doctor is better than a computer – can look at context/broader picture | Considering context of readings. | “So I ring her and I say, “xxx, your blood pressure’s too high this month.” She goes, “Yes, I know, I’ve had a stressful time, my father-in-law’s unwell.” We discussed, “Do you want to increase your blood pressure medication?” She’s like, “No, everything’s fine, he’s going into a home, things are going to calm down.” So the computer doesn’t have the flexibility to really look at the entire situation” (Prescriber 5) |
|  |  | Waiting another month for more evidence of a need for a change | Perceiving that more evidence is needed over a longer period of time to change medication | “Here should be possibly a sort of a watchful, waiting period rather than reacting to just a fairly short term trend in the blood pressure” (Prescriber 5) |
|  |  | Patient’s readings were borderline | Considering the thresholds for changing medication in HOME BP | “And actually when you look at the statistics it’s one systolic point below the threshold and what I would say was probably perfectly well controlled and yet the Home BP is just looking at it in black and white ways” (Prescriber 5) |
|  |  | Not relying on home readings | Prescriber questions whether they can rely on home readings to change medication. | “I normally do six readings myself here, just to make sure sort of it's, you know, coinciding with their readings… Sometimes when I've done it the readings have been quite different..... I suppose because I can see what's happening, I can see that the cuff's on properly, I can see that they're relaxed etc. And I guess because I see the readings that are right in front of me” (Prescriber-supporter 3) |
|  |  | Considering lifestyle changes rather than medication escalation | Reluctance to escalate medication without trying lifestyle change. | “To consider whether there’s any lifestyle changes that need implementing in the short to medium term, rather than just going for medication changes at each stage” (Prescriber 5) |
|  |  | Wanting to check adherence to current medication | Perceptions about low adherence to medication acting as a barrier to medication escalation | “Then we talk about their medication, make sure they're actually taking their medication because I've had a few people who, you know, when you actually question them they've forgotten to take it or they've not been taking it as regularly as they should've been” (Prescriber-supporter 3) |
|  |  | Unnecessary stress or hassle for patient | Concerns about the stress or hassle recommendations for medication escalation could have caused their patients. | “I guess one of them, the patient found – I think frustrating because it's – it clearly pushed them into being more alarmed than perhaps they should have been” (Prescriber 7) |
|  |  | Some patients need less blood pressure medication | Where patients’ medication has been reduced during the study. | “And we've managed to down titrate patients on medication because of their, you know, the white coat syndrome if you like” (Supporter 8) |
