# Supplementary material for: Implementing a digital intervention for managing uncontrolled hypertension in Primary Care: a mixed methods process evaluation
Source: Implement Sci. 2021 May 26;16:57. doi: 10.1186/s13012-021-01123-1 (PMC8152066; doi:10.1186/s13012-021-01123-1)
Supplement: Supplementary file 7 — Additional file 7. [file 13012_2021_1123_MOESM7_ESM.docx]

**Additional file 7. Adherence rates for target behaviours**

Table S4. Spearman’s correlations between prescriber questionnaire measures pre-training and post-training, and adherence to intervention behaviours

| Questionnaire variables | | Pre or post-training | Prescriber adherence to planning 3 medication escalations  (n=67) | Prescriber adherence to initiating recommended medication escalations within 28 days  (n=59) | Prescriber adherence to changing medication remotely (n=50) |
| --- | --- | --- | --- | --- | --- |
| Prescriber self-efficacy | a. Create individualised patient medication plans | Pre-training | 0.22 | 0.41** | 0.19 |
|  |  | Post-training | 0.17 | 0.26* | 0.34* |
|  | b. Increase patient medication when blood pressure remains too high | Pre-training | 0.14 | 0.22 | 0.22 |
|  |  | Post-training | 0.13 | 0.31* | 0.27 |
|  | c. Integrate the HOME BP programme into regular care | Pre-training | -0.15 | 0.31* | 0.08 |
|  |  | Post-training | -0.03 | 0.20 | 0.20 |
| Prescriber outcome expectancies mean score | | Pre-training | -0.11 | 0.13 | -0.08 |
|  |  | Post-training | -0.04 | 0.08 | -0.22 |
| Prescriber perceived acceptability of the intervention for patients | a. Self-monitor their blood pressure at home | Pre-training | 0.15 | -0.03 | 0.17 |
|  |  | Post-training | -0.09 | -0.02 | 0.01 |
|  | b. Enter their blood pressure readings into HOME BP | Pre-training | -0.04 | 0.18 | 0.16 |
|  |  | Post-training | -0.15 | 0.03 | 0.07 |
|  | c. Make medication changes to control their blood pressure | Pre-training | 0.25 | 0.25 | 0.23 |
|  |  | Post-training | -0.10 | -0.11 | 0.16 |

**p* < 0.05, ** *p*<0.001

Table S5 Spearman’s correlations between supporter questionnaire measures pre-training and post-training, and adherence to intervention behaviours

| Questionnaire variable pre-training | Supporter adherence to sending monthly emails (n=62) | |
| --- | --- | --- |
|  | Pre-training | Post-training |
| Supporter self-efficacy mean | -0.11 | -0.19 |
| Supporter outcome expectancies mean | -0.13 | -0.15 |
| Supporter confidence in patients mean | 0.11 | -0.02 |

P<.05

Table S4. Contextual patient factors and Practitioner adherence to medication escalation recommendations (n=405)

| Variable | Cases of adherence | Cases of non-adherence | *Test statistic* | *Effect size* |
| --- | --- | --- | --- | --- |
| Mean systolic blood pressure reading | 140.0 mmHg | 136.7 mmHg | *t= -4.10* | *d = 0.41* |
| Mean diastolic blood pressure reading | 84.3 mmHg | 82.9 mmHg | *t= -1.92* | *d = 0.19* |
| Mean age of patient | 63.6 years | 62.6 years | *t= -1.22* | *d = 0.12* |
| Median n of monthly blood pressure entries | 4 | 6 | *U= 14127* | *r^2^ = 0.07* |
| Median n of medication escalation recommendations already received for patient in question | 2 | 3 | *U=14018* | *r^2^ = 0.08* |

Chi-squared tests revealed no difference between prescribers’ adherence to medication escalation for patients with lower blood pressure targets due to diabetes and patients with standard targets (χ^2^ (2, N = 403) = 1.16, *p*>.05). Patients aged over 80 years with adjusted targets could not be included due to small sample size (n=2).
